# Investigating the effectiveness of different aspirin dosing regimens and the timing of aspirin intake in primary and secondary prevention of cardiovascular disease: protocol for a systematic review

**DOI:** 10.1186/s13643-015-0078-3

**Published:** 2015-06-19

**Authors:** Danai Bem, Janine Dretzke, Simon Stevens, Marie Lordkipanidzé, James Hodgkinson, Sue Bayliss, David Moore, David Fitzmaurice

**Affiliations:** 1 Public Health, Epidemiology and Biostatistics, School of Health and Population Sciences, College of Medical and Dental Sciences, University of Birmingham, Edgbaston, Birmingham, B15 2TT UK; 2 NIHR Surgical Reconstruction and Microbiology Research Centre, Queen Elizabeth Hospital Birmingham, Edgbaston, Birmingham, B15 2TH UK; 3 Montreal Heart Institute, Research Centre, 5000 rue Bélanger, Montréal, QC H1T 1C8 Canada; 4 Faculté de pharmacie, Université de Montreal, C.P. 6128, Succ. Centre-ville, Montreal, QC H3C 3J7 Canada; 5 Primary Care Clinical Sciences, School of Health and Population Sciences, College of Medical and Dental Sciences, University of Birmingham, Edgbaston, Birmingham, B15 2TT UK

**Keywords:** Aspirin, Aspirin doses, Split dosing, Aspirin regimens, Timing, Primary prevention, Secondary prevention, Cardiovascular disease, Systematic review

## Abstract

**Background:**

Once-daily low-dose aspirin is routinely used for the prevention of secondary events in cardiovascular disease (CVD). The routine use of aspirin in primary prevention of CVD is less clear due to a finer balance between benefits and harms. In addition, the variability in benefit achievable from the prescription of aspirin has led to a growing interest in considering whether there are more effective aspirin regimens than once-daily dosing or whether effectiveness is influenced by the time of day aspirin is taken (chronotherapy). The proposed systematic review will evaluate the evidence on the effects of different aspirin regimens used in terms of number of doses (e.g. split or alternate dosing) or dosing time of aspirin (e.g. morning versus evening) in primary and secondary prevention of CVD.

**Methods/design:**

Standard systematic review methodology will be employed for study identification, selection and data extraction. Electronic databases will be searched incorporating terms relating to population and the intervention. No date or language limitations will apply. Systematic reviews and controlled studies comparing different aspirin regimens—in terms of frequency or timing—for primary and/or secondary prevention of CVD will be included. No restrictions on outcome will apply. Quality assessment will be appropriate for each study design. The data will be tabulated and narratively synthesised. Meta-analysis may be undertaken where clinical and methodological homogeneity exists.

**Discussion:**

There are a number of published and ongoing primary studies that investigate the cardiovascular protective effect of different aspirin regimens. However, no systematic review to date has attempted to review the evidence pertaining to aspirin dosing regimens differing in frequency and/or in timing. The proposed systematic review will cover both the above questions and could potentially be beneficial for reconsidering the current practice of managing patients with aspirin in primary care.

**Systematic review registration:**

PROSPERO CRD42014010596

**Electronic supplementary material:**

The online version of this article (doi:10.1186/s13643-015-0078-3) contains supplementary material, which is available to authorized users.

## Background

Cardiovascular disease (CVD) continues to be the leading clinical and public health problem worldwide, causing 17.5 million deaths each year [1]. Aspirin is one of the most common, useful and inexpensive antiplatelet drugs used for the prevention of CVD.

In line with the UK and EU guidelines [2, 3], patients with established CVD are recommended long-term treatment with daily low-dose aspirin that effectively reduces the yearly risk of serious adverse events such as myocardial infarction, stroke, or vascular death. For secondary prevention of CVD, the benefits of daily low-dose aspirin therapy substantially exceed the risks [4]. There is considerable uncertainty though around the benefits of aspirin in individuals with no history of prior CVD but who may be at risk of cardiovascular events [5]. To that end, health organisations recommend the use of aspirin for primary prevention of CVD when a net benefit is present [6]. Indeed, some evidence suggests a small prophylactic effect from daily low-dose aspirin in patients with cardiovascular risk factors, but any potential clinical benefit should be carefully weighed against a potential increase in the risk of undesirable effects (e.g. gastrointestinal bleeding and haemorrhagic stroke) [7, 8].

It is known that aspirin therapy is not equally effective in all patients, with some experiencing repeat cardiovascular events despite aspirin administration. Poor compliance with the treatment may be one explanation for less effective aspirin therapy [9, 10]. Variable response to aspirin may also depend on the patient population studied, patient age, co-morbidities and/or co-medications, and kinetics of aspirin targets [11, 12]. Recent evidence has suggested that accelerated platelet function recovery after inhibition with aspirin could be an additional factor for this observed variability in platelet responsiveness [13–18].

It has been suggested that altering the standard dosing regimen of once-daily aspirin (e.g. to twice-daily) or changing the timing of aspirin intake may confer better cardiovascular protection in some patient populations. Chronotherapy, an emerging concept in the field of therapeutics, is a treatment method in which administration of a drug is timed to match the circadian rhythm of disease in an attempt to optimise the therapeutic outcome and minimise treatment side effects [19]. The recommended aspirin regimen is one tablet (75–150 mg) per day to be taken at least 30 min before breakfast. However, it is known that the incidence of thromboembolic events has a peak period during the morning [20]. Taking aspirin in the morning will result in inhibition of platelet function occurring within 60 min of ingestion [21] and may result in highest plasma level of the drug being reached after the morning peak incidence of cardiovascular events. It has therefore been proposed that evening intake of aspirin may result in a more protective effect [17, 22].

In addition, the potential merit of a split-dosing regimen (e.g. twice- versus once-daily) has been reported in patients with diabetes [17, 23–25] or hypertension [26]. Based on these studies, it has been suggested that patients with suboptimal response to aspirin or those where aspirin treatment appears to have been ineffective may benefit from this dosing regimen as a result of a greater platelet inhibition or reduction in blood pressure. In contrast, less frequent administration of aspirin (e.g. every other day) could be an alternative regimen in order to minimise adverse events, such as bleeding, from long-term treatment, though it is currently not known in which patient groups this might be beneficial.

Despite the existence of a number of systematic reviews that focus on the effectiveness of daily low-dose aspirin in primary and secondary prevention of CVD [4, 5, 7], a scoping search in MEDLINE and The Cochrane Library identified no existing systematic reviews that focus on both frequency of dosing and timing of aspirin administration. A broad review with some systematic methodological elements was identified [19], which covered the chronotherapy (i.e. timing of therapy) of a range of drugs, including aspirin (searches up to 2011). There were some methodological limitations to this review, including language and date restrictions and a lack of quality assessment of included studies. The review was also not reported in accordance with the Preferred Reporting Items for Systematic Reviews and Meta-Analyses (PRISMA) guidelines. Thus, the robustness of the overall findings is uncertain. A further systematic review and meta-analysis from 2011 on timing of administration was identified [27], however, this appears to be published in abstract form only, and full details on methodology could not be ascertained. Scoping searches also indicate that a number of recent publications that are likely to be relevant are not included in the above reviews. Thus, an up-to-date, methodologically robust, systematic review is warranted. The proposed systematic review will evaluate the evidence on the effects of different aspirin regimens (in terms of number of doses and/or timing of aspirin administration) used in primary and secondary prevention of CVD.

## Methods/design

### Research questions

The aim is to undertake a systematic review of the evidence on alternative dosing and timing regimes of aspirin used in primary and secondary prevention of CVD. This can be split in two research questions:

- The effect of split daily dosing (e.g. once versus twice or more) or alternate-day dosing of aspirin compared to a single daily dose on primary and secondary prevention of CVD, and
- The effect of timing of aspirin intake (e.g. evening versus morning) on primary and secondary prevention of CVD

This protocol is registered with PROSPERO (CRD42014010596) and has been reported here according to the PRISMA-P guidelines (Additional file 1). The project was presented to a patient group in order to help inform the protocol.

### Searches

The following sources will be searched for primary studies:

- Bibliographic databases—MEDLINE, MEDLINE In Process and EMBASE via Ovid, CINAHL via EBSCO, and The Cochrane Library (CDSR, DARE and CENTRAL databases)
- Science Citation Index (Web of Science) for citation searching
- The ISRCTN database, UKCRN, WHO ICTRP Portal and ClinicalTrials.gov for ongoing studies
- Specialist abstract and conference proceeding resources (British Library’s ZETOC and Conference Proceedings Citation Index (Web of Science))
- Checking of citation lists of included studies and relevant reviews
- Selected websites of organisations such as the British Heart Foundation (BHF), the National Institute for Health and Care Excellence (NICE) and the American Heart Association (AHA)

A combination of text words and MeSH terms relating to number of doses and timing of aspirin will be utilised. Given the variable terminology for these elements, the search strategies will be broad using a range of terms, such as once/twice-daily, once/twice a day, alternate-day or split-dosing, morning/awakening versus evening/bedtime, time of taking, daily administration, chronopharmacology, chronoefficacy etc. There will be no restriction on date or language of publication. A sample search strategy for MEDLINE is provided in Additional file 2, and this strategy has been adapted for use in each bibliographic database.

The literature search results will be entered onto EndNote X7.1 (Thomson Reuters, New York) to facilitate removal of duplicate records, study selection, recording decisions and referencing. The study selection process will be conducted in two stages: (1) title and abstract screening and (2) full-text reading of potentially relevant studies. At both stages, two reviewers will independently assess the studies for inclusion using predetermined eligibility criteria. Any discrepancies between reviewers will be resolved by discussion, and in case of disagreement, a third reviewer will be involved until consensus is reached. Translation of non-English language articles will be undertaken if necessary. The selection process will be illustrated using a PRISMA study flow chart.

### Selection criteria

#### Study design

A preliminary scoping search suggested that the volume of primary studies is likely to be small. To this end, any controlled studies (randomised or non-randomised, prospective or retrospective, concomitant or historical control) will be included. Non-controlled studies, single case reports and narrative reviews will not be considered.

#### Participants

Participants will be individuals given aspirin for primary or secondary prevention of cardiovascular events. Primary prevention includes patients with diabetes, hypertension or another disease (like atrial fibrillation) with a potentially high risk for a cardiovascular event to occur. Secondary prevention includes patients with established cardiovascular disease. Patients in an acute setting where aspirin is administered post-operatively to prevent thromboembolic events, such as deep vein thrombosis, pulmonary embolism and embolic stroke, will be considered but as a distinct group. Studies with mixed population will be included providing results for a population of interest can be extracted. Pregnant women at risk of developing gestational hypertension or preeclampsia and studies on healthy volunteers will be excluded. Observational studies reporting different levels of incidental or self-selected aspirin use (i.e. not prescribed for primary or secondary prevention) will also be excluded. There will be no age restrictions.

#### Intervention and comparator

Aspirin dosing regimens will include the following:

- Split-dosing: aspirin taken twice or more per day compared to a regimen with lower frequency (e.g. once per day)
- Alternate-day dosing: aspirin taken every other day compared to a more frequent regimen (e.g. every day)
- Different times of the day, e.g. aspirin taken in the morning compared to the evening

The focus will be on aspirin as the sole antiplatelet therapy (monotherapy), but studies where same additional therapies are given in all study arms will also be included (e.g. dual or triple therapy). Where there is a difference in both overall dose and number of doses or timing, the study will be included. However, if the only difference is the dose (e.g. different doses taken daily and at the same time), the study will be excluded.

#### Outcomes

There will be no restriction by outcome. Based on scoping searches, there are likely to be only a small number of studies reporting clinical and/or patient-related outcomes of interest. Therefore, surrogate end points will also be included.

Primary outcomes

- Cardiovascular events (e.g. myocardial infarction, ischemic stroke, transient ischemic attack composite outcomes, e.g. MACE (major adverse cardiac events))
- Mortality
- Adverse events (e.g. bleeding)

Secondary outcomes

- Blood pressure
- Platelet function as measured with a platelet function test
- Compliance

There will be no restriction of length of study or type of effect measure.

### Data extraction

Data extraction will be conducted by one reviewer using a standardised, piloted data extraction form, with selected data, particularly relating to quality and outcomes, checked by a second. Discrepancies will be resolved through discussion or referral to a third reviewer. The following information (but not limited to) will be extracted from all included studies:

- Study characteristics: country of origin, study design, setting, sample size, length of follow-up
- Population: patient inclusion and exclusion criteria, co-morbidities, co-medications, platelet kinetics
- Intervention/comparator: dose, timing of dose, number of doses, patient adherence (how assessed/reported)
- Results: completeness of follow-up, outcome measures, statistical methods employed, findings, effect sizes and associated uncertainty

Study authors and researchers may be contacted if further information is required.

### Quality assessment

Assessment of all selected publications for their methodological quality will be undertaken. Study quality of primary studies will be assessed using tools specific to each study design. The risk of bias tool from the Cochrane Handbook [28] will be used to assess the internal validity of randomised and non-randomised controlled trials, considering different types of bias such as selection, performance, detection, attrition and reporting bias. The quality of each article will be appraised by two reviewers independently, and the reasons for their decisions will be recorded. Disagreements will be resolved by discussion or referral to a third reviewer. Should relevant cross-over trials be identified, then additional areas of risk of bias will need to be assessed, e.g. whether there is a carry-over effect due to insufficient washout periods between phases, whether only relevant first period data are available, and comparability of results with those from parallel-group trials.

For controlled observational studies, the domains in the risk of bias tool for RCTs will be used as a minimum quality assessment (regardless of the fact that there is no randomisation). The most relevant criteria for assessment are likely to relate to how the groups were selected, differences in patient characteristics, loss to follow-up, and biases in outcome assessment and reporting. In addition, the extent to which confounders have been given adequate consideration, both in reporting and analysis, will be examined. For historical control studies, additional factors to be included are possible changes in the diagnostic criteria, outcome definitions/measurements and/or differences in concomitant standards of care over time.

The quality information will be used in the context of considering the robustness of evidence for each outcome for each comparison, as well as indicating any common issues for consideration when designing any new studies.

### Analysis

Synthesis of evidence will be undertaken separately for the different systematic review questions. Narrative synthesis of evidence will be conducted for all included studies with the extracted data to be tabulated as defined in the data extraction section. It is most likely that the outcome measures will be expressed as continuous data (e.g. mean/median or mean difference in blood pressure or platelet aggregation) or, for cardiovascular events, as dichotomous data.

Meta-analysis will be conducted where appropriate to combine data from similar studies. Assessment of clinical and methodological heterogeneity will be used to determine whether meta-analysis is appropriate and if so, whether a fixed effect or random effects model should be used. Statistical heterogeneity will be measured by using Higgins and Thompson’s *I*^2^ and chi-squared statistics [29]. Evidence from RCTs and other controlled studies will not be quantitatively combined together, but presented separately (both on a study and outcome level). Visual representation of results in forest plots without pooling may also be considered.

Sub-group analysis will be considered where studies can be grouped according to patient characteristics (e.g. those taking aspirin for primary or secondary prevention) or overall dose. The robustness of results to methodological quality of included studies may be explored in sensitivity analysis; this will be dependent on sufficient reporting of quality criteria in order to make an overall assessment of study quality. The potential for the existence of publication bias will be assessed via funnel plots if there are at least 10 studies in a given meta-analysis, and using the appropriate statistical tests for small study effects (such as the Peters Test [30]).

### Reporting

The Grading of Recommendations, Assessment, Development and Evaluation (GRADE) framework [31] will be used to summarily consider inconsistency (or heterogeneity) between studies, precision (uncertainty) of results, likelihood of publication bias and applicability of results to population(s) of interest. The review and its findings will be reported in accordance with the PRISMA guidelines [32].

## Discussion

A large number of people take a daily low-dose aspirin tablet for prophylaxis against prospective or recurrent cardiovascular events. The benefit seen from such therapy is however variable between patient groups and individuals, and it has been proposed that changes to dosing regimens (frequency or timing of aspirin administration) may be beneficial to some in terms of improving the efficacy of aspirin. An up-to-date systematic review of the evidence on different dosing regimens therefore appears to be warranted. Results from the review will need to be interpreted in the context of study quality, documented adherence of patients to aspirin therapy and relevance of the outcome measures used in the included studies. Further, the use of aggregate data (particularly of continuous outcomes) may mean that findings are not necessarily generalisable to individual patients. Nonetheless, findings are likely to be important given the large number of patients on aspirin currently being managed in primary care. Any gaps in the existing evidence will be highlighted in order to inform future research directions including new trials in this area.

## Additional files

Additional file 1:
**PRISMA-P checklist.**
Additional file 2: **Sample search strategy.** Sample search strategy to identify relevant primary studies in MEDLINE.

## Abbreviations

CVD: cardiovascular disease
RCT: randomised controlled trials
