# Supplementary material for: Investigating the effectiveness of different aspirin dosing regimens and the timing of aspirin intake in primary and secondary prevention of cardiovascular disease: protocol for a systematic review
Source: Syst Rev. 2015 Jun 19;4:88. doi: 10.1186/s13643-015-0078-3 (PMC4475616; doi:10.1186/s13643-015-0078-3)
Supplement: Additional file 2: — Sample search strategy. Sample search strategy to identify relevant primary studies in MEDLINE. [file 13643_2015_78_MOESM2_ESM.pdf]

Additional file 2

Database: Ovid MEDLINE(R) <1946 to September Week 4 2014>

Search Strategy:

1 (twice adj2 (day or daily)).mp. [mp=title, abstract, original title, name of substance word, subject heading word, keyword heading word, protocol supplementary concept word, rare disease supplementary concept word, unique identifier] (43793)

2 (once adj2 (day or daily)).ti,ab. (36498)

3 daily administrat$.ti,ab. (5557)

4 or/1-3 (77654)

5 exp Aspirin/ (38799)

6 acetylsalicylic acid.ti,ab. (7147)

7 ASA.ti,ab. (16794)

8 aspirin.ti,ab. (35635)

9 or/5-8 (68035)

10 4 and 9 (1052)

11 (aspirin adj4 (regime$ or timing or time or circadian or evening or morning or alternate or split or frequency or "every other" or night$ or bedtime$ or awakening or waking or chrono$)).ti,ab. (981)

12 10 or 11 (1971)

13 exp Cardiovascular Diseases/ (1915641)

14 exp Coronary Artery Disease/ (39128)

15 exp Diabetes Mellitus/ (322380)

16 exp Cerebrovascular Disorders/ (285128)

17 exp Hypertension/ (214204)

18 exp Thrombocythemia, Essential/ (2318)

19 heart disease$.ti,ab. (120817)

20 diabetes.mp. or diabetic.ti,ab. [mp=title, abstract, original title, name of substance word, subject heading word, keyword heading word, protocol supplementary concept word, rare disease supplementary concept word, unique identifier] (439948)

21 coronary artery disease$.ti,ab. (58103)

22 thrombocythemia.ti,ab. (2676)

23 (cerebrovascular adj (disease$ or disorder$)).ti,ab. (15218)

24 hypertension.ti,ab. (268810)

25 blood pressure.ti,ab. (215817)

26 stroke.mp. or strokes.ti,ab. [mp=title, abstract, original title, name of substance word, subject heading word, keyword heading word, protocol supplementary concept word, rare disease supplementary concept word, unique identifier] (179902)

27 or/13-26 (2436265)

28 12 and 27 (1059)

29 limit 28 to humans (992)
